# Quantitative Lipidome Analysis of Boiled Chicken Egg Yolk under Different Heating Intensities

**DOI:** 10.3390/molecules28124601

**Published:** 2023-06-07

**Authors:** Wei Luo, Jinghui Wang, Yan Chen, Qionglian Zhang, Jinqiu Wang, Fang Geng

**Affiliations:** 1 Institute for Egg Science and Technology, School of Food and Biological Engineering, Chengdu University, Chengdu 610106, China; 2 Fengji Food Group Co., Ltd., Mianyang 621603, China

**Keywords:** egg yolk, lipidome, triglycerides, phospholipids, heating

## Abstract

The effects of the four heating intensities (hot-spring egg yolk, HEY; soft-boiled egg yolk, SEY; normal-boiled egg yolk, NEY; and over-boiled egg yolk, OEY) on lipidomes of boiled egg yolks were investigated. The results indicated that four heating intensities had no significant effect on the total abundance of lipids and lipid categories except for bile acids, lysophosphatidylinositol, and lysophosphatidylcholine. However, of all the 767 lipids quantified, the differential abundance of 190 lipids was screened among the egg yolk samples at four heating intensities. Soft-boiling and over-boiling altered the assembly structure of the lipoproteins through thermal denaturation and affected the binding of lipids and apoproteins, resulting in an increase in low-to-medium-abundance triglycerides. The decreased phospholipid and increased lysophospholipid and free fatty acid in HEY and SEY suggests potential hydrolysis of phospholipids under relatively low-intensity heating. Results provide new insights into the effect of heating on the lipid profiles of egg yolk and would support the public’s choice of cooking method for egg yolks.

## 1. Introduction

Eggs contain all the nutrients needed for the development of the chicken embryo and are considered a nutritionally balanced food that is easily digested and absorbed [1,2]. Owing to their low consumption cost and high nutritional value, eggs are the preferred food for low-income groups to improve dietary nutrition and are also a staple food of middle- and high-income groups. Consumers have multiple demands in terms of taste quality, nutritional safety, and convenience. Therefore, to meet the requirements of different consumers, numerous egg processing methods emerged [3,4]. Heat treatment is the most common method of cooking eggs, which includes the boiling, poaching, scrambling, steaming, and frying of eggs. Heat processing not only denatures egg proteins to improve their digestion and absorption rate, but also effectively controls harmful microorganisms, enhancing the safety of ingested eggs [1,5,6,7]. However, heat processing inevitably leads to changes in the nutrients and qualities of eggs [8]. Therefore, there is a need to evaluate the heating conditions that have the lowest impact on egg nutrients and are more conducive to digestion and absorption.

Previous studies focused more on the effects of heat treatment on egg whites, especially on egg white allergens [9,10,11,12]. However, egg yolks are more diverse in terms of nutritional composition, especially their rich lipid content, which is also sensitive to heat processing [13]. Recent studies found that intense heat processing may result in the loss of the thermally unstable vitamins A and E in the egg yolk, as well as potentially producing some substances (e.g., FeS) that are detrimental to digestion and absorption [1,14]. However, egg yolk lipid molecule species are diverse, and hundreds of species were identified based on the optimized lipidome analysis workflow [15]. Furthermore, in previous studies, our group used lipidomics techniques to quantify the changes in lipid fractions during yolk formation and to further compare the differences in the lipid composition of egg yolk, yolk granules, and yolk plasma [16,17]. In a previous study, the extraction solvent used in metabolomics was water–methanol (70:30, *V*:*V*) in order to more comprehensively detect multiple species of metabolites (e.g., amino acids, etc.) in the samples; 104 lipids were eventually detected, and it was found that over-boiling treatment may lead to hydrolysis of egg yolk phospholipids [18]. Therefore, the systematic understanding of the changes in egg yolk lipid profiles during heating remains inadequate. 

Boiling is one of the most common home-cooking methods for eggs. Different heating intensities (different heating temperature and time) have significant effects on the sensory properties of egg yolks, including egg yolk texture, color, smell, and swallowability. However, the effects of heating intensity on yolk lipid molecules were not systematically investigated. Therefore, in this study, yolks subjected to different heating treatments, including fresh egg yolk (FEY), hot-spring egg yolk (HEY), soft-boiled egg yolk (SEY), normal-boiled egg yolk (NEY), and over-boiled egg yolk (OEY), were used to compare the effects of heating intensity on the texture and surface color of the yolk. In addition, quantitative lipidome analysis was used to explore the effects of different heating intensities on the lipid molecules in egg yolk. The results of this study will provide systematic data for understanding the changes in the nutrients present in egg yolk during cooking.

## 2. Results

### 2.1. Egg Yolk Surface under Different Heating Intensities

**Figure 1:**
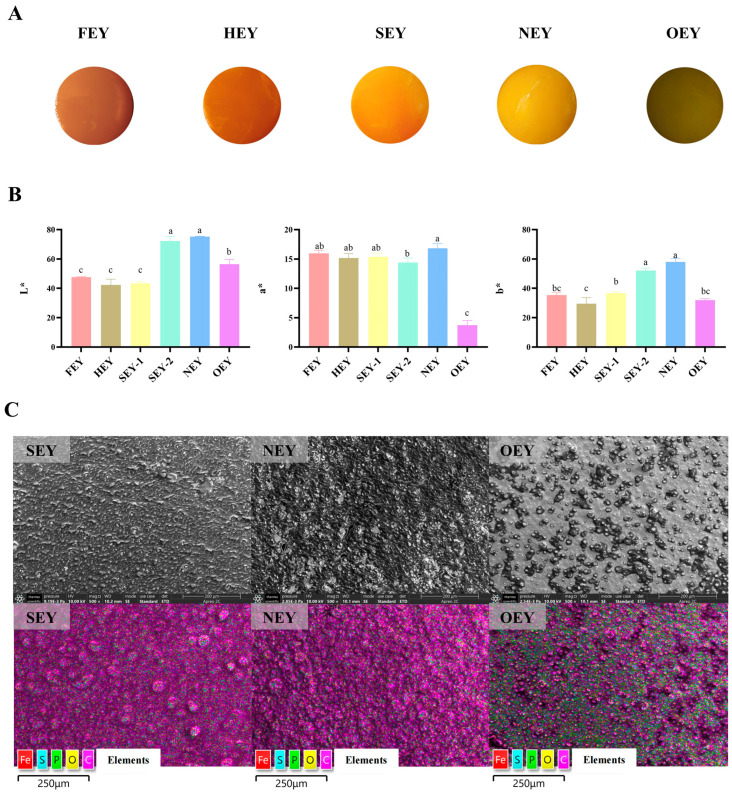
Effect of different-intensity heat treatments on the color and micromorphology of egg yolk surface. (**A**) Images of the surface of egg yolks. (**B**) L*, a* and b* values of egg yolk surfaces. (**C**) SEM images and major element distribution on the surface of egg yolks. Significant differences (*p* < 0.05) between six groups are indicated by different letters (a–c).

The surface of the egg yolk was observed after heat treatment at different intensities (Figure 1A). Both the FEY and HEY were fluid-like and close to dark orange color, while the NEY was completely solidified and showed a distinctly different bright yellow surface. In particular, the SEY was partially solidified and two colored areas appeared on its surface. The color of the unsolidified area was close to the surface color of the HEY, and the color of the solidified area was close to the surface color of the NEY. The OEY surface appeared greenish–gray. The results of the chromameter measurements corresponded to the results observed (Figure 1B). Among them, the FEY and HEY surfaces showed lower L* (lightness) values and b* (yellowness) values, while the NEY surface showed higher *L* values. The two regions of the surface of the SEY showed very different L* and b* values. Compared with the NEY, the OEY surface showed lower L*, a* (redness), and b* values, suggesting that the OEY was darker, greener, and bluer in color. This color of the OEY deviated from the color of normally cooked egg yolk and would cause unpleasant sensory perception to consumers.

The SEM results showed differences in the surface microstructure of the SEY, NEY, and OEY (Figure 1C). Among them, the SEY surface was mainly linear in structure and initially started to form a mesh structure. The NEY surface formed a homogeneous mesh-like porous structure, while the OEY surface further agglomerated or “hardened”, forming lumpy structures of different sizes. An EDS was used to compare the major elemental contents and their distribution on the surfaces of the SEY, NEY, and OEY (Figure 1C). Compared with the SEY and NEY, the carbon and oxygen content on the surface of the OEY differed significantly, and the percentage of oxygen increased from 13.47% (FEY) and 14.08% (NEY) to 20.44% (OEY). Other elements such as iron, sulfur, and phosphorus were not detected because they were below the detection limits.

### 2.2. Textural Properties of Egg Yolk under Different Heating Intensities

**Figure 2:**
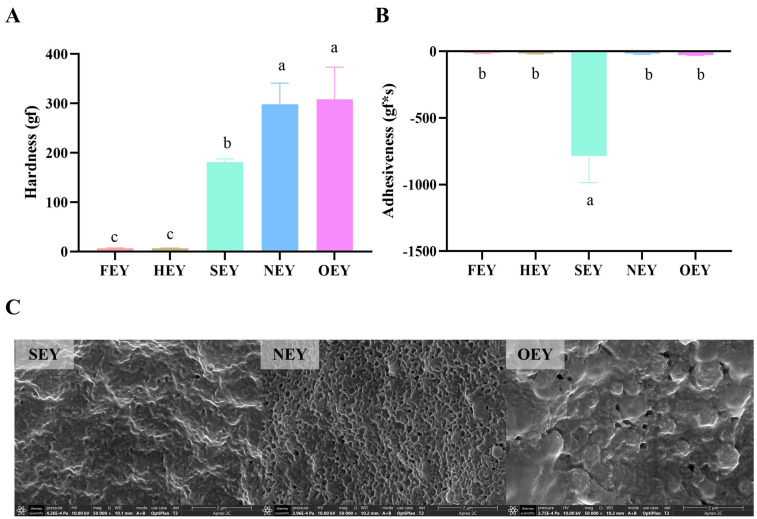
Effect of different-intensity heat treatments on the taste and texture of the egg yolk. (**A**) Effect of different-intensity heat treatments on the hardness of egg yolks. (**B**) Effect of different-intensity heat treatments on the adhesiveness of egg yolks. (**C**) SEM images of the internal microstructure of SEY, NEY, and OEY. Significant differences (*p* < 0.05) between five groups are indicated by different letters (a–c).

The TPA results showed that the hardness of the egg yolk samples increased gradually with increasing heating intensities (Figure 2A). The hardness of the SEY was significantly different from that of the other four yolks and was an intermediate transitional form between the unformed (FEY and HEY) and fully solidified (NEY and OEY) egg yolks. For adhesiveness, the FEY and HEY showed less adhesiveness because they were flowing solutions; the NEY and OEY were hard solids and, therefore, also less adhesive to the probe (Figure 2B). However, the SEY showed stronger adhesiveness, mainly because it was in a semi-solid state.

Comparing the SEM images of the internal sections of the SEY, NEY, and OEY, the SEY initially started to coagulate internally and tended to form an ordered linear structure (Figure 2C). The NEY was further condensed inside, forming a mesh-like porous structure. In addition, as the heating intensity increased, the internal condensation of the OEY formed a hardened structure and the overall orderliness was disrupted.

### 2.3. Effect of Different Heat Treatment Intensities on the Egg Yolk Lipidome

Egg yolk lipids have a variety of nutritional and biological effects. The systematic detection and evaluation of yolk lipids and contents at different heating intensities will be useful to understand the changes in the nutritional value of egg yolk.

#### 2.3.1. Overview of the Lipidome of Egg Yolk

**Figure 3:**
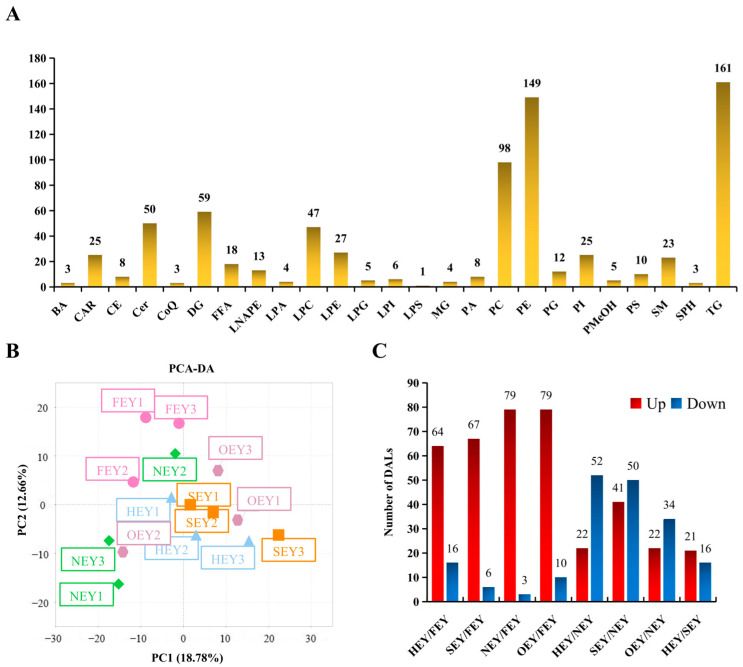
Lipidomic identification of egg yolks under different heating intensities. (**A**) Number of lipid molecules contained in the lipid species identified in FEY, HEY, SEY, NEY, and OEY. (**B**) Principal component analysis (PCA). (**C**) The number of differentially abundant lipids (DALs) in the pairwise comparison between groups.

Firstly, the sample quality control (QC) results demonstrated the high stability of the instruments and assays used in this study, which also provided important assurance of the reproducibility and reliability of the data from subsequent lipidomics analyses (Figure S2). In this study, 767 lipids were identified in five groups of egg yolk samples: FEY, HEY, SEY, NEY, and OEY (Table S2). These lipids belonged to 25 lipid categories, mainly including 161 triglycerides (TGs), 149 phosphatidylethanolamines (PEs), 98 phosphatidylcholines (PCs), 59 diglycerides (DGs), 50 ceramides (Cers), 47 lysophosphatidylcholines (LPCs), 27 lysophosphatidylethanolamines (LPEs), 25 phosphatidylinositols (PIs), 25 carnitines (CARs), 23 sphingomyelins (SMs), and 18 free fatty acids (FFAs) (Figure 3A).

#### 2.3.2. Overall Differences in Yolk Lipidomes under Different Heating Intensities

The lipidome profiles of the FEY, HEY, SEY, NEY, and OEY were compared using principal component analysis (PCA) and orthogonal partial least squares discriminant analysis (OPLS-DA). The overall differences in the yolk lipidome under different heating intensities were assessed. PCA analysis showed that the FEY samples were separated from the other groups of samples on the PCA score plots, indicating the effect of heating on the lipidome of egg yolk (Figure 3B). However, the egg yolk samples were not clearly separated on the PCA score plots after heating, which suggests that the overall difference in the yolk lipidome under different heating intensities was not significant. Further pairwise comparisons were performed using OPLS-DA. The results showed 21.2–28.2% for component 1 and 14.8–28.7% for component 2 in the pairwise comparisons (Figure S3), indicating that the differences in the yolk lipidome between groups could be resolved using OPLS-DA.

#### 2.3.3. Changes in the Abundance of Lipid Categories

The abundance of the 25 lipid categories in egg yolk under different heating intensities was calculated (Figure S4). The results showed that the abundance of bile acids (BA) and lysophosphatidylinositol (LPI) in the HEY was significantly lower (*p* < 0.05) than that in the OEY. The other 23 lipid categories varied in abundance but did not differ significantly. These results were consistent with the PCA analysis where different heating intensities did not cause substantial changes in the abundance of egg yolk lipids. Therefore, a more detailed analysis of the data is required, and differences in abundance at the lipids level need to be further screened.

#### 2.3.4. Differential Abundance of Lipids (DALs)

Based on the variable importance in projection (VIP; VIP ≥ 1) from the OPLS-DA, and combined with a fold change (FC; FC > 1.5 or FC < 0.67) in lipid abundance, a total of 190 DALs were screened in pairwise comparisons of the five egg yolk lipidomes. Among them, the abundance of 13 DALs changed significantly in all four egg yolk samples after heating compared with the FEY; these were FFA (22:3), FFA (20:4), FFA (24:5), FFA (20:3), FFA (20:2), FFA (18:2), FFA (20:1), FFA (16:0), FFA (22:5), PC (14:0_15:0), TG (14: 1_18:2_18:2), carnitine C12:1, and coenzyme Q9. These 13 DALs could serve as potential markers of heated egg yolks.

HEY is a minimally heated egg yolk that did not yet reach the thermal denaturation of egg yolk proteins. Nonetheless, 80 DALs (64 up and 16 down) were found in the HEY compared with the FEY (Figure 3C). These DALs mainly included 17 FFAs (17 up), 13 TGs (13 up), 9 Cers (9 up), 8 LPCs (8 up), 8 PIs (8 down), 5 PEs (3 up, 2 down), and 5 LPEs (5 up). The increased abundance of FFAs may suggest potential lipid hydrolysis in HEY. 

SEY is an increasingly loved and popular edible form of egg yolk. Compared with the FEY, the abundance of 73 (67 up and 6 down) lipids changed significantly in the SEY. These DALs mainly included 30 TGs (30 up), 13 DGs (13 up), 12 FFAs (12 up), 5 PCs (5 up), 4 Pes (3 up, 1 down), and 4 Pis (4 down). The increased abundance of a larger number of TG species is an interesting result, and the underlying causes are proposed in the Section 3 (Discussion Section). In the comparison of the SEY/NEY, 91 DALs (41 up 50 down) were screened. They mainly included 17 TGs (16 up, 1 down), 12 DGs (12 up), 11 LPEs (4 up, 7 down), 10 LPCs (1 up, 9 down), 9 phosphatidylserines (PSs, 9 down), and 6 PIs (6 down).

NEY is an egg yolk with the most commonly accepted heating strength by consumers. In the NEY/FEY comparison, 82 (79 up, 3 down) DALs were screened. They mainly included 16 FFAs (16 up), 14 LPCs (14 up), 13 LPEs (11 up, 2 down), 9 TGs (9 up), 9 PSs (9 up), 5 PEs (5 up), 4 lysophosphatidic acids (LPAs, 4 up), and 3 phosphatidic acids (PAs, 3 up).

OEY is more commonly seen in large-scale dining; for example, canteens of schools and companies, fast food chains, and group catering for large events. In the OEY, 89 (79 up, 10 down) DALs were screened in comparison with the FEY. These DALs mainly included 23 TGs (23 up), 14 FFAs (14 up), 13 LPCs (12 up, 1 down), 10 LPEs (8 up, 2 down), 8 PCs (8 up), 6 LPIs (6 up), and 4 PSs (4 down). There were 56 (22 up, 34 down) DALs in the OEY compared with the NEY, mainly including 9 PSs (9 down), 9 PEs (9 down), 8 TGs (6 up, 2 down), 5 PCs (5 up), and 5 PAs (5 down).

#### 2.3.5. Changes in the Most Abundant Lipids

**Figure 4:**
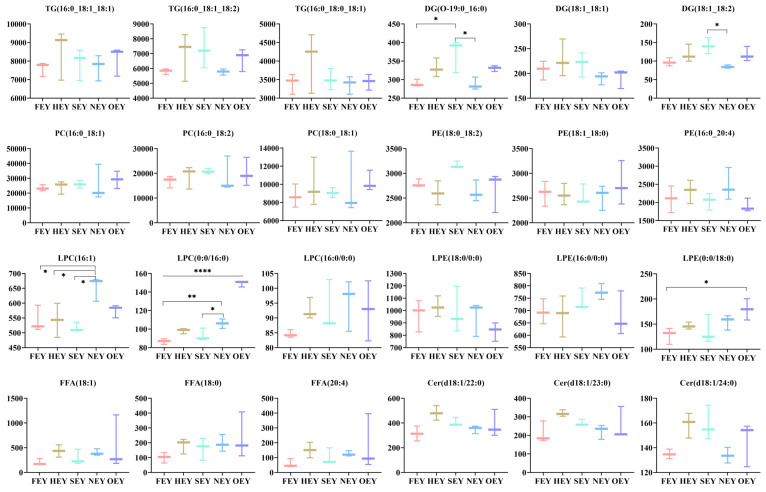
The content of the three most abundant lipid molecules of the eight lipid categories TG, DG, PC, PE, LPC, LPE, FFA, and Cer in egg yolk under different heating intensities. Significant differences between groups are indicated by different numbers of asterisks (*, *p <* 0.05; **, *p <* 0.01; ****, *p <* 0.0001).

The egg yolk content of the three most abundant lipid molecules of the eight lipid categories, TG, DG, PC, PE, LPC, LPE, FFA, and Cer, under different heating intensities was compared using FEY as the control (Figure 4). It was found that the more abundant lipid molecules in several major yolk lipid categories, such as TG and DG, were richer in fatty acids with a carbon chain length of 16/18, suggesting that the related fatty acids (C16/18) are important constituent fatty acid molecules in the major egg yolk lipids. In addition, the abundance of some major lysophospholipid molecules increased at higher heat treatment intensities. Comparing the FEY, HEY, and SEY, the abundance of LPC (16:1) increased significantly in the NEY (*p* < 0.05). The abundance of LPC (0:0/16:0) was significantly higher in the NEY and OEY than in the FEY (*p* < 0.05), and the abundance of LPE (0:0/18:0) was also significantly higher in OEY (*p* < 0.05). However, the abundance of DG (O-19:0_16:0) and DG (18:1_18:2) decreased significantly in the NEY compared with the SEY (*p* < 0.05).

#### 2.3.6. The Representative DALs

**Figure 5:**
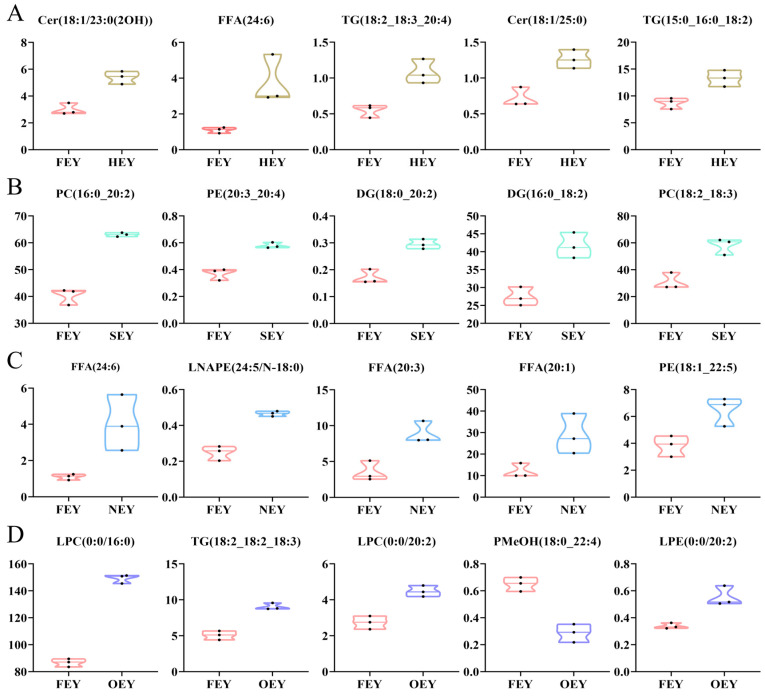
The top five differential abundance lipids (DALs) with VIP values in the four intensity heat-treated egg yolks compared to FEY ((**A**), HEY/FEY, (**B**), SEY/FEY, (**C**), NEY/FEY, (**D**), OEY/FEY).

The five lipid molecules that contributed most to the differences in lipidomes (highest VIP values) in the HEY compared with the FEY included two Cers (Cer(18:1/23:0(2OH), Cer(18:1/25:0)), two TGs (TG(18:2_18:3_20:4), TG(15:0_16:0_18:2)), and one FFA (FFA(24:6)), and their abundances were all higher in the HEY (Figure 5A). With increasing heating intensity, the five lipids with the highest VIP values in SEY/FEY were three glycerophospholipids and two diglycerides (Figure 5B), indicating that the changes in the yolk lipid group caused by different heating intensities were different. In the NEY/FEY comparison, the three FFAs, LNAPE (24:5/N-18:0), and PE (18:1_22:5) with higher abundance in the NEY were the most representative (Figure 5C). Among the overheated OEY, the three lysophospholipids with higher abundance than the FEY were the most representative DALs (Figure 5D).

## 3. Discussion

### 3.1. Heating Affects the Color and Micromorphology of the Egg Yolk Surface

As an important criterion in the sensory evaluation of food, the color of food directly affects the choice and eating preference of the consumer [18,19]. In this study, significant differences were found in the surface color of egg yolks with different intensities of heat treatment. This was consistent with previous findings which highlighted that when the heating temperature exceeded 75 °C, the surface color of the egg yolk changed significantly [20]. Carotenoid pigments (lutein; including lutein, zeaxanthin, b-cryptoxanthin, and small amounts of b-carotene) are the main substances responsible for the coloration of egg yolks. However, it was found that ordinary heat treatment has little effect on the lutein content of egg yolks [21].

The denaturation of egg yolk protein caused by heating and the resulting change in the organization and interaction of egg yolk nutrients should be the main reasons for the color change in egg yolks. During the gel formation process of egg yolks, the change in lipid distribution seriously affects the distribution of the coloring substances in the yolk, which affects the internal and surface color. It was found that the color of the yolk changes with the migration of lipids within the yolk during the salting process [22]. This might be because the color-presenting substances, such as lutein, in egg yolks are mostly lipid-soluble, and the distribution state of different color-presenting substances may vary with the lipid. Moreover, the physical state of the egg yolk also affects the surface color. The results of this study showed that the liquid phase before gel formation had a significantly different L* value compared with the solid phase after gel formation. Therefore, the distribution and gelation state of the coloring substances within the yolk may be an important reason for the significant difference in HEY and NEY colors and the in-between color of SEY. Egg yolk, as a nonhomogeneous solution system, is composed of suspended particles (yolk particles) and protein macromolecules (plasma), and solute migration occurs during the heating-induced phase transition, which also affects the yolk color [23].

The oxidation of lipids and protein can also affect the color of egg yolks. Heat treatment was found to induce the oxidation of egg yolk lipids and proteins in a previous study [13]. The oxidation of lipids and proteins in egg yolk during salting leads to an increase in pyrrole pigment concentration and nonenzymatic browning; this leads to a decrease in yolk a* and b* values [24]. This might partly explain the significant decrease in the OEY surface a* values and b* compared with the SEY and NEY.

It was speculated in the literature that the surface blackening of OEY may be due to the combination of iron ions in the egg yolk with the sulfur ions produced by heating the protein to ferrous sulfide [14]. However, no abnormal enrichment of sulfur and iron on the surface of the egg yolk was found in this study. This may be related to the detection limit of the EDS, which needs to be further explored. In addition, the carbon/oxygen ratio was downregulated, indicating an elevated oxygen ratio on the yolk surface, but the specific causes and effects of the elevated oxygen ratio remain to be investigated subsequently.

The surface blackening of egg yolk not only affects the sensory evaluation of the yolk, but may also reduce the absorption and use of nutrients in the yolk [14]. Therefore, further studies are needed to uncover the mechanisms underlying the blackening of the surface of egg yolks. Additionally, during actual cooking and production, the heat treatment temperature and time should be fully considered to avoid overcooking and the blackening of the surface of egg yolks.

### 3.2. Heating Affects the Taste and Texture of the Egg Yolk

As another key indicator used to characterize the sensory properties of food, the texture of food reflects, to a certain extent, the real oral perception of food during chewing. The findings were obtained in this study, where the intensity of heat treatment severely affected the hardness and adhesiveness of the egg yolk. In the process of converting an egg yolk from a liquid to a solid under heat treatment, that is, in the process of forming yolk gel, various physicochemical interactions occur between the components of the egg yolk [25]. Some studies reported that the aggregation of low-density lipoprotein (LDL) and yolk particles in egg yolk begins to occur at 65–70 °C; however, at this point, a continuous gel was not found to have started to form [26,27]. Therefore, the HEY is still in a fluid form and did not start to form a gel. Furthermore, during the folding and unfolding of lipoproteins due to heat denaturation, small amounts of lipids are released, which fill the yolk gel structure, and these lipids further increase the yolk adhesiveness [23,28].

When the temperature of the heat treatment is further increased, the interaction between LDL and the yolk granule components intensifies and initially induces the formation of protein gels [26]. The enhanced heating conditions can also expose the sulfhydryl groups inside the protein and promote the formation of disulfide bonds, resulting in tighter protein cross-linking and further enhancing the gel strength [29]. This is the reason for the increasing hardness from SEY to OEY. These findings were further verified by the observation of microstructure changes on the surface and fractured sections of the egg yolk. That is, the linear structure of the SEY implies greater adhesiveness, while the homogeneous mesh-like porous structure of the NEY represents increased hardness. Similar findings were reported where heat treatment initially induced lipoproteins to form linear fibrillar aggregates, followed by further fibrillar cross-linking [30,31]. For the disruption of the ordered structure in the OEY, it is speculated that the heating time may be too long, leading to the disruption of the internal structure of the egg yolk, which tends to be disordered. In contrast, it may be that the migration of lipids within the yolk intensifies, thus breaking the ordered cross-linked structure formed relative to the fully cooked stage. For example, it was found that heat treatment leads to the release and migration of lipids, resulting in the formation of lamellar structures in the high-fat region of egg yolks [23].

### 3.3. Heating Might Affect the Detectability of Egg Yolk Lipids

**Figure 6:**
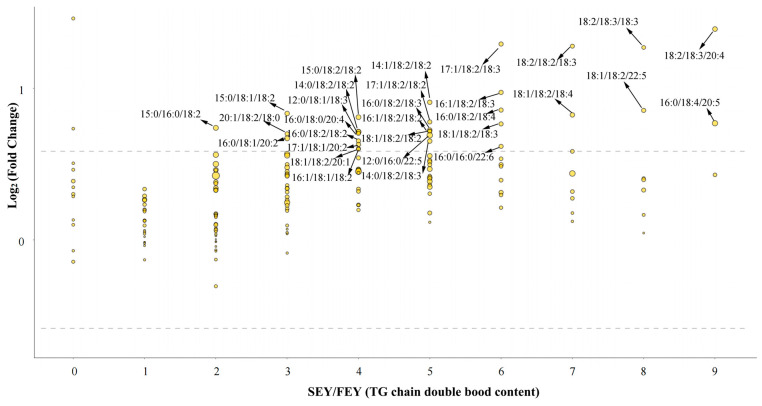
The number of double bonds contained in the 30 differential TG molecules in SEY/FEY.

In addition to the abovementioned factors, differences in extraction solvents may affect the final amount of lipids detected [18]. Lipids in egg yolk are in the form of lipoproteins [32], which means that the affinity between lipids and apoproteins can affect the extraction of lipids in the preprocessing of the sample and, in turn, affect their detectability in the subsequent lipidome analysis. This speculation was mainly supported by data illustrating that there was an increased abundance of TGs in the heated egg yolk samples. Quantitative analysis showed that the abundance of the top three TG molecules did not change significantly in the heat-treated egg yolk, which indicates the high stability of TGs during thermal processing (Figure S5, Table S2). However, the abundance of some medium-abundance and low-abundance TGs changed significantly. There were 13, 30, 9, and 23 types of TGs that showed significantly increased abundance in the HEY, SEY, NEY, and OEY, respectively. A further in-depth analysis found that the high-abundance TG species that did not change significantly had low unsaturation (containing 0–2 double bonds); furthermore, the low-abundance TG species that changed significantly had high unsaturation (containing 3–9 double bonds) (Figure 6 and Figure S5). Theoretically, it is unlikely that TGs would be newly synthesized in egg yolks through an enzymatic catalytic process or a chemical reaction process during the heating process. Therefore, the increase in the abundance of these low- and medium-abundance TGs was more likely to be the result of the reorganization of yolk lipoproteins during heating denaturation. 

The TGs in egg yolk are mainly in the form of LDL, as a lipid core wrapped by a phospholipid monomolecular layer [27]. Heat treatment causes denaturation and structural remodeling of LDL, resulting in a change in the state or presence of TGs, which makes it easier for them to be extracted with solvents (methyl tert-butyl ether and methanol mixture, 3:1, *v/v*). This may be the main reason for the significant increase in the abundance of some TG molecules in egg yolk due to heating. It was also reported that the structure of LDL particles in egg yolk is disrupted under treatment at 60–70 °C and above, thus inducing more lipids to be freed [13,33]. This further explains the difference in the number of differentially abundant TGs in the HEY/FEY and SEY/FEY. Moreover, as the intensity of the heat treatment increases, the gel networks formed in the NEY and proteins tend to aggregate in an orderly manner, and these changes limit the migration and distribution of lipid molecules. A study reported that the aggregation products of LDL contain lipids internally, and that the amount of lipids extracted from heat-treated LDL decreases with increasing temperature when the treatment temperature is above 75 °C [34]. However, with more prolonged heat treatment, which leads to disruption of the gel network, the protein aggregation state is altered and the movement of lipid molecules is further intensified. This may be the reason for the appearance of more differentially abundant TG molecules in the OEY/FEY. Therefore, the reasons for the increased abundance of TG molecules in higher-intensity heating may be influenced by various factors, such as yolk lipoprotein denaturation and chemical reaction processes, which require further investigation. Heating may also significantly affect the gastrointestinal digestion and absorption of egg yolk TGs by altering the molecular state of egg yolk TGs, but this also requires investigation. It was found that the structure and composition of TG molecules at the oil/water interface in emulsions during digestion seriously affect the hydrolytic absorption efficiency of TG molecules [35,36].

### 3.4. Heat-Induced Phospholipid Hydrolysis in HEY and SEY

**Figure 7:**
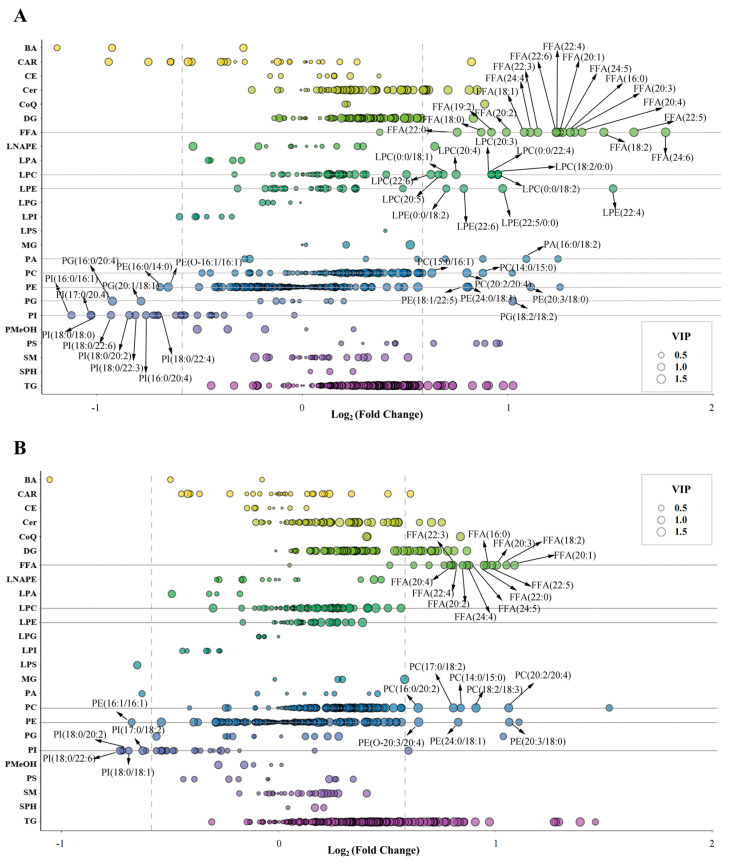
Scatter plots of the main differential lipid distributions in HEY/FEY (**A**) and SEY/FEY (**B**).

The potential hydrolysis of some phospholipids in egg yolk (OEY) induced by heat treatment and the potential increase in the abundance of some FFAs were initially identified in previous metabolomics studies; however, due to the small number of lipids detected by metabolomics (only 55 “glycerophospholipids” and 49 “fatty acyls”), the systematic effects of different intensities of heat treatment on yolk lipids could not be fully demonstrated [18]. In this study, heat treatment significantly decreased the abundance of various phospholipids in the HEY and SEY, and significantly increased the abundance of lysophospholipids, PAs, and FFAs. Among them, a significant decrease in the abundance of 12 phospholipids and a significant increase in the abundance of 12 lysophospholipids, 9 Cers, 1 PA, and 17 related FFAs were observed in the comparison of HEY/FEY (Figure 7A). Similarly, a significant decrease in the abundance of five phospholipids and a significant increase in the abundance of 2 Cers and 12 FFAs were found in the comparison of SEY/FEY (Figure 7B). These data suggest the potential hydrolysis of phospholipids in HEY and SEY.

Egg yolks are rich in endogenous lipases, including acid lipase, neutral lipase, and phospholipase [37,38]. Among them, the optimum temperature of egg yolk phospholipase is 40–55 °C [39,40]. Therefore, the phospholipid hydrolysis may be accelerated in the temperature ramp-up period during the heating process, which is more likely to occur in HEY and SEY with a lower heating intensity. The endogenous enzyme-induced hydrolysis reaction resulted in a significant decrease in the abundance of some phospholipids in HEY and SEY, and a significant increase in the abundance of some corresponding FFAs and lysophospholipids. Similar results were also reported in stored egg yolks: the abundance of FFAs increased while the abundance of PI and PE decreased with storage time [37]. By comparing the changes in phospholipid degradation levels in HEY and SEY, it was also found that higher heating temperatures and faster ramping rates might reduce the time of endogenous phospholipase and, thus, reduce phospholipid hydrolysis. Lipid hydrolysis induced by endogenous lipase was also reported in other foods during heating. For example, phospholipases in pork and cow’s milk have a higher activity below 60 °C, which induces more phospholipids to undergo hydrolysis [41,42]. 

With a further increase in heat treatment intensity, some FFA abundances in the NEY and OEY also showed a significant increase. This may be caused by the breakdown of some lipid molecules induced by a high-intensity heat treatment rather than the hydrolysis of endogenous lipases. In a previous study on the extraction of oils and fats from herring by-products using heat treatment, the FFA content in herring oil obtained under treatment at 60–90 °C tended to decrease and then increase, with 70 °C being the inflexion point [43]. Therefore, 70 °C is likely to be junction point between endogenous lipases, especially phospholipase, inactivation, and the thermal hydrolysis of lipids.

### 3.5. Effects of Heating on the Nutritional Value of Egg Yolk Lipids

**Figure 8:**
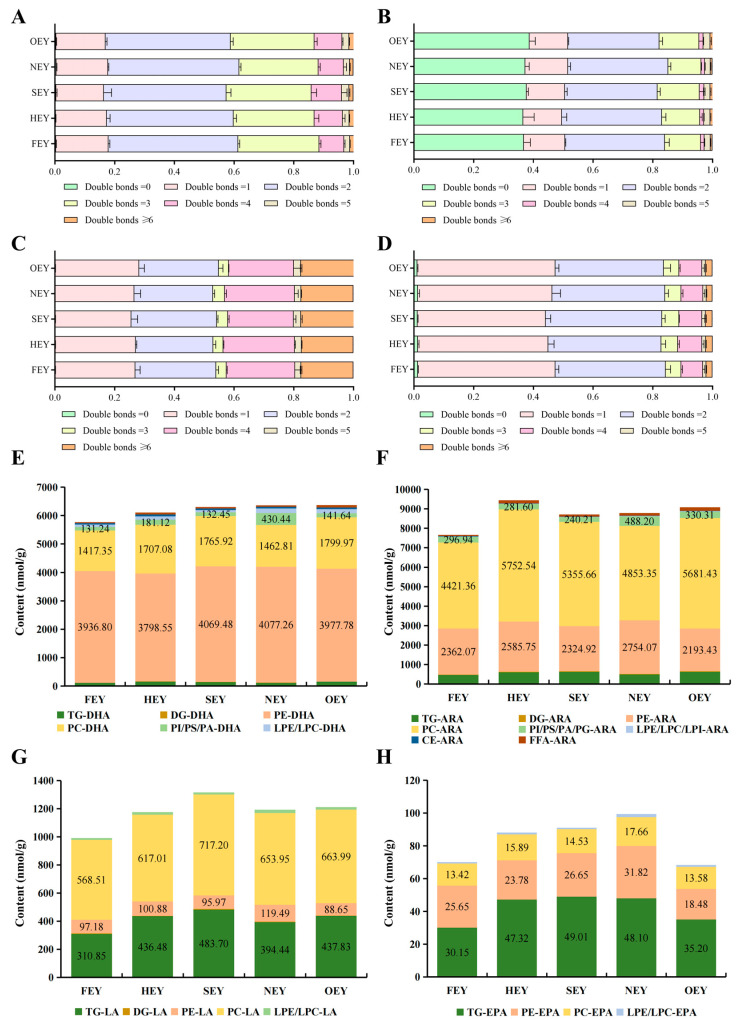
Percentage abundance of lipids by number of unsaturated double bonds in egg yolks heat-treated to different intensities ((**A**), TG; (**B**), DG; (**C**), PE; (**D**), PC) and comparison of the abundance of lipid species containing DHA (C22:6), ARA (C20:4), LA (C18:3), and EPA (C20:5) ((**E**), DHA; (**F**), ARA; (**G**), LA; (**H**), EPA). The horizontal coordinate is the percentage abundance based on mass spectral signal intensity of lipid species.

The number of double bonds in lipids is considered an important indicator of the antioxidant capacity of lipids and is also used as an important reference for evaluating their nutritional value [44]. Therefore, in this study, the number of unsaturated double bonds of TG, DG, PE, and PC in egg yolks at different heating intensities was calculated and compared (Figure 8A–D). It was found that the proportions of double bonds in the heated egg yolks were similarly distributed without significant differences. Among them, the percentages of lipid molecules containing two or more double bonds in TG, DG, PE, and PC were 82.06–83.11%, 48.42–50.43%, 71.79–74.42%, and 52.62–55.90%, respectively. Therefore, TG and PE are richer in polyunsaturated fatty acid molecules than DG and PC. However, the high proportion of unsaturated double bonds also increases the risk of oxidation during processing or storage to a certain extent. For example, a previous study showed that the PE in egg yolk is more susceptible to lipid oxidation than the PC during storage [37].

Some polyunsaturated fatty acids in egg yolks are of considerable interest because of their high nutritional value, including docosahexaenoic acid (DHA), eicosapentaenoic acid (EPA), linolenic acid (LA), and eicosatetraenoic acid (ARA) [45,46]. In this study, 54 lipids containing DHA, 12 lipids containing EPA, 26 lipids containing LA, and 68 lipids containing ARA were identified (Table S3). In a further comparative analysis, the total amount of lipids containing fatty acid fractions, such as DHA, ARA, and LA, in the heated egg yolks were also not significantly different (*p* > 0.05) (Figure 8E–G). In addition, polyunsaturated fatty acids are mainly present in egg yolks in the form of phospholipids and triglycerides; however, heat treatment affects the form in which these polyunsaturated fatty acid molecules exist (Figure 8E–H). After heat treatment, the percentage of PE-DHA in the egg yolk decreased (from 68.27% to 62.25–64.63%), and the corresponding percentage of other phospholipid forms of DHA molecules increased. Furthermore, the percentage of PC-LA in the yolk decreased after heat treatment (from 57.33% to 52.50–54.83%), and conversely, the percentage of TG-LA increased (from 31.35% to 33.07–37.14%). Similarly, the percentage of TG-EPA in the yolk increased after heat treatment (from 43.02% to 48.38–53.86%), while this corresponded to a decrease in the percentage of PE-EPA (from 36.60% to 26.98–32.00%). The form in which lipid molecules containing ARA exist also undergoes a complex change. Thus, although the total amount of lipid molecules containing DHA, ARA, and LA does not change significantly after heat treatment, the form in which these polyunsaturated fatty acids exist is altered accordingly, which will affect the efficiency of their digestion and absorption by the human gastrointestinal tract and metabolism. For example, DHA and EPA in the form of phospholipids are more beneficial to human metabolism than those in the form of triglycerides [47,48]. However, the exact effect requires further study.

## 4. Materials and Methods

### 4.1. Sample Preparation

Fresh eggs were provided by Fengji Food Technology Co., Ltd. (Mianyang, China). The eggs were heated in different modes (different heating temperature and time) using a common household egg boiler (ZDQ-B06R2, Xiaoxiong Electric Co., Ltd., Foshan, China). The rise in temperature based on the heating program is shown in Figure S1 [the total heating time for the HEY was 22 min, with the temperature gradually increasing from room temperature to 68.5 °C (at 13 min) and then stabilizing. The total heating time for the SEY was 14 min, i.e., a gradual increase from room temperature to 96 °C (at 11 min) and then stabilization. The NEY was heated for a total of 17 min, gradually increasing from room temperature to 96 °C (at 11 min) and then stabilizing. The OEY was heated for a total of 60 min and was also gradually heated from room temperature to 96 °C (at 11 min) and then stabilized]. The heated eggs were soaked in 2 L plain deionized water at 20–25 °C for 1 min. When the eggs were removed from the water, they were placed in an empty 2 L beaker to cool to 40 °C. Then, the egg shells were immediately removed and the whites separated to collect the egg yolks.

### 4.2. Color Measurement

The images were captured from the yolk surface using a smartphone (Honor 30, Zhixin New Information Technology Co., Ltd., Shenzhen, China) to observe heat-treated yolk discoloration at the interface between the yolk and egg white according to the unaided eye [14]. In addition, the color of the yolk surface was measured using a chromameter (CR9, Sanenshi Technology Co., Ltd., Shenzhen, China). Where the L* value indicates the brightness of the sample (0–100 indicates from black to white), the a* value indicates the red-greenness color of the sample (positive values indicate redness, negative values indicate greenness) and the b* value indicates the yellow-blueness color of the sample (positive values indicate yellowness, negative values indicate blueness).

### 4.3. Texture Profile Analysis (TPA)

The whole egg yolk was placed into a 100 mL beaker and then mashed and flattened. The texture profile analysis of the egg yolk was conducted using a texture analyzer (TA. Touch, Baosheng Industrial Development Co., Ltd., Shanghai, China) with a TA/0.5 probe (0.5 inch diameter). The test parameters were test speed: 1.0 mm/s; interval time: 5 s; target mode selection displacement: 10 mm; and contact point type selection pressure: 10 gf.

### 4.4. Micromorphology Characterization

The surface samples of the SEY, NEY, and OEY were taken, and they were vacuum freeze-dried (−80 °C for 36 h, LC-12N–80C, Lichen Bonsi Instrument Technology Co., Ltd., Shanghai, China). The micromorphology of the egg yolk surface was observed using a scanning electron microscope (SEM, Thermo scientific Apreo 2C, Thermo Fisher Scientific, Shanghai, China). In addition, the main elements of the egg yolk surface were determined using an energy dispersive spectrometer (EDS, Oxford Ultim Max65, Oxford Instruments Technology, Oxford, UK). The typical images were selected for presentation.

The SEY, NEY, and OEY were fractured and lyophilized, and the fractured sections of the yolk samples were observed using SEM.

### 4.5. Lipidomic Analysis

#### 4.5.1. Sample Preparation

Based on the method in Ref. [49] and improved, seven egg yolks were randomly selected from each heating intensity and mixed well. Then, 20 mg of each yolk sample was accurately weighed and put into a 2 mL centrifuge tube. Next, 1 mL of lipid extraction solution (methyl tert-butyl ether and a methanol mixture containing an internal standard, 3:1, *v*/*v*) was transferred into the sample tube, and steel beads were added to the tube; the sample solution was then homogenized and mixed well. After, the steel beads were removed, and the tube was vortexed and shaken for 2 min and sonicated for 5 min; then, 200 μL of water was added, followed by continuous vortexing for 1 min. Subsequently, the tube was centrifuged at 15,160× *g* for 10 min at 4 °C, and 200 μL of the supernatant was transferred into a new 1.5 mL centrifuge tube; the solvent was then evaporated using a vacuum concentrator (CentriVap, Labconco, Kansas City, MO, USA). The extracted samples were re-solubilized with 200 μL of lipid dissolving solution (acetonitrile solution containing 0.04% acetic acid) for subsequent LC-MS/MS analysis. The total lipid extraction of each set of egg yolk samples was repeated three times, and the lipid extracts from each replicate were separately subjected to subsequent LC-MS/MS analysis.

#### 4.5.2. LC-MS/MS

The data acquisition instrument system consisted primarily of ultra-performance liquid chromatography (UPLC) (ExionLC™ AD, https://sciex.com.cn/) and tandem mass spectrometry (MS/MS) (QTRAP^®^ 6500+, https://sciex.com.cn/).

The column used for the liquid chromatography separation was Thermo Accucore™ C30 (2.6 µm, 2.1 mm × 100 mm i.d.). Mobile phase A was acetonitrile/ultrapure water (60/40, *v*/*v*) (containing 0.1% formic acid and 10 mmol/L ammonium formate); mobile phase B was acetonitrile/isopropanol (10/90, *v*/*v*) (containing 0.1% formic acid and 10 mmol/L ammonium formate). The separation was carried out in a gradient system: 0 min for A/B (80:20, *v*/*v*), 2 min for A/B (70:30, *v*/*v*), 4 min for A/B (40:60, *v*/*v*), 9 min for A/B (15:85, *v*/*v*), 14 min for A/B (10:90, *v*/*v*), 15.5 min for A/B (5:95, *v*/*v*), 17.3 min for A/B (5:95, *v*/*v*), 17.5 min for A/B (80:20, *v*/*v*), and 20 min for A/B (80:20, *v*/*v*). The injection volume was 2 µL, the column temperature was 45 °C, and the flow rate was 0.35 mL/min.

The temperature of the electrospray ionization was 500 °C. The mass spectrum voltage was 5500 V in the positive ion mode and −4500 V in the negative ion mode. Ion source gas 1 (GS1) was set to 45 psi, GS2 was set to 55 psi, and curtain gas (CUR) was set to 35 psi. In the triple quadrupole, each ion pair was scanned based on the optimized declustering potential and collision energy.

#### 4.5.3. Qualitative and Quantitative Analysis

Based on the self-built database MWDB (more than 3000 lipid molecules, Metware Biotechnology Co., Ltd. Wuhan, China), qualitative analysis was carried out according to the retention time (RT) of the detected substances, the information of the parent–child ion pair, and the secondary spectrum data. In addition, the CAS number, KEGG pathway, and other supporting annotation information were determined, thus improving the accuracy of the qualitative analysis of the substance. Multiple reaction monitoring (MRM) mode analysis of triple quadrupole mass spectrometry was utilized in the lipid quantification. After obtaining the data from the lipid mass spectrometry analysis of the different samples, the peak areas of the mass spectral peaks of all substances were integrated and quantified using the internal standard method. The internal standards used in this study are shown in Table S1. The actual concentration (abundance) of the substance in the sample was then calculated from the area ratio of the peak areas of all samples detected by the internal standard method, using the following formula:

X = 0.001 × R × c × F × V/m

where X is the amount of lipid in the egg yolk sample (nmol/g), R is the ratio of the peak area of the substance to be measured to that of the internal standard, F is the correction factor for internal standards of different types of substances, c is the concentration of the internal standard (μmol/L), V is the volume of the sample extraction solution (μL), and m is the mass of the egg yolk sample (g).

### 4.6. Statistical Analysis

All measurements were performed in triplicate and the results were expressed as the mean plus with standard deviation. Statistical analysis was carried out by one-way analysis of variance using GraphPad Prism 8.0 software, with *p* < 0.05 indicating significant differences. Multivariate statistical analyses, including principal component analysis (PCA) and orthogonal partial least squares discriminant analysis (OPLS-DA), were further carried out [50]. In this study, PCA was performed with the built-in statistical prcomp function of the R software (www.r-project.org/), setting the prcomp function parameter scale = true to indicate unit variance scaling of the data. OPLS-DA was log_2_ transformed on the raw data and then centralized, where X is the sample quantitative information matrix and Y is the sample grouping information matrix. It was then analyzed using the MetaboAnalystR package OPLSR.Anal function in the R software. In addition, the OPLS-DA evaluation model used in this study was subjected to 200 randomized permutations of the data.

## 5. Conclusions

In this study, with increasing heat treatment temperature (68.5–96 °C) and time (14–60 min), the internal structure of the yolk changed from linear to mesh-like porous to lumpy structure, which, in turn, led to an increasing overall hardness of the yolk. Among them, over-boiling treatment can cause a greenish–gray egg yolk coloration and an increase in egg yolk hardness, which can affect the sensory acceptability of the food. In contrast to previous metabolomics, which only found that over-boiling treatment leads to hydrolysis of egg yolk phospholipids, the quantitative lipidomic results showed that certain intensities of heat treatment brought about changes in the status and distribution of some yolk lipids, such as an easier extraction of unsaturated TG molecules in the SEY, hydrolysis of some phospholipid molecules in the HEY and SEY, and a decrease in the abundance of lipid molecules containing the EPA (C20:5) fraction in the OEY. Heat treatment changed the form of some polyunsaturated fatty acids, such as DHA, LA, and EPA that were present. Therefore, a given intensity of heat treatment may potentially affect the digestion and absorption of egg yolk nutrients by the human intestine; however, further studies are needed. Finally, this study provided a theoretical reference for the scientific selection of cooking methods for egg yolks.

## Data Availability

Not applicable.

## Supplementary Materials

The following supporting information can be downloaded at: https://www.mdpi.com/article/10.3390/molecules28124601/s1, Figure S1: Heating temperature versus time curves during the preparation of HEY, SEY, NEY, and OEY. Figure S2. Results of quality control (QC) sample analysis in the egg yolk lipidomic assay. (A) Total ion current (TIC) plot for mass spectrometry analysis of the QC sample of the same mass in positive-ion mode. (B) TIC plot for mass spectrometry analysis of the QC sample of the same mass in negative-ion mode. (C) Pearson correlation analysis of QC samples. (D) Distribution of coefficient of variation (CV) values of QC samples. Figure S3: Orthogonal partial least squares discriminant analysis (OPLS-DA) of the lipid profile of egg yolk heat-treated at different intensities. The values in parentheses are the frequencies of occurrence of random grouping models that outperformed the predictive power (Q^2^) of this OPLS-DA model and the interpretation of the Y matrix (R^2^Y), respectively, in 200 random permutation experiments. Figure S4: Changes in the abundance of lipid categories in egg yolk with different intensity of heat treatment. Significant differences (*p* < 0.05) between five groups are indicated by different letters (a–b). Figure S5: Number of double bonds contained in differentially abundant TG molecules in HEY, NEY, and OEY (A, HEY/FEY; B, NEY/FEY; C, OEY/FEY). Table S1: The internal standards used in this study. Table S2: Lipid identification information for all samples. Table S3: Content of lipid molecules containing polyunsaturated fatty acid components in heat-treated egg yolks of different intensities.
