# Supplementary material for: Quantitative Lipidome Analysis of Boiled Chicken Egg Yolk under Different Heating Intensities
Source: Molecules. 2023 Jun 7;28(12):4601. doi: 10.3390/molecules28124601 (PMC10301504; doi:10.3390/molecules28124601)
Supplement: Supplementary file 1 [file molecules-28-04601-s001.zip › Supporting materials-Figures.pdf]

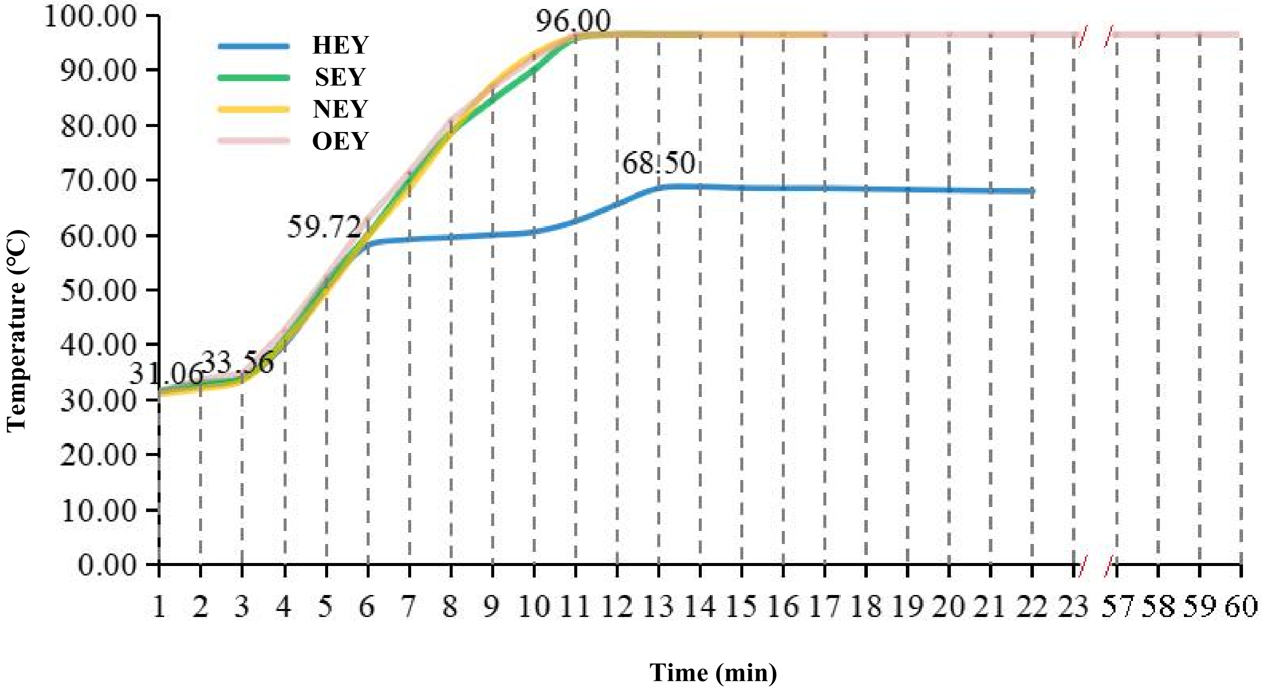

Figure S1: Heating temperature versus time curves during the preparation of HEY, SEY, NEY and OEY.

Figure S2. Results of quality control (QC) sample analysis in the egg yolk lipidomic assay. (A) Total ion current (TIC) plot for mass spectrometry analysis of the QC sample of the same mass in positiveion mode. (B) TIC plot for mass spectrometry analysis of the QC sample of the same mass in negative-ion mode. (C) Pearson correlation analysis of QC samples. (D) Distribution of coefficient of variation (CV) values of QC samples.

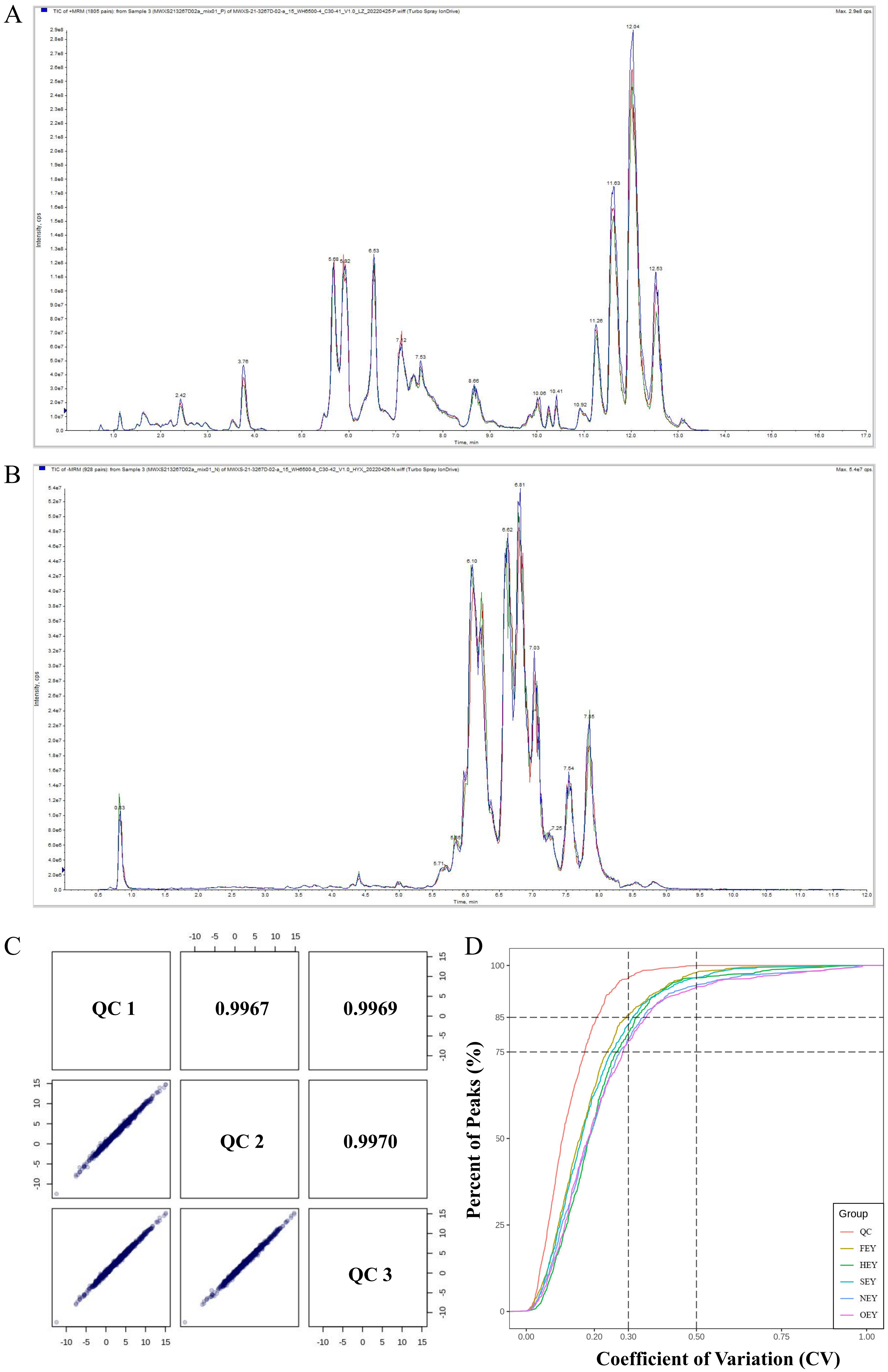
A
Intensity, cps
Time, min
B
Intensity, cps
Time, min
C
QC 1
0.9967
0.9969
QC 2
0.9970
QC 3
D
Percent of Peaks (%)
Coefficient of Variation (CV)
Group
QC
FEY
HEY
SEY
NEY
OEY

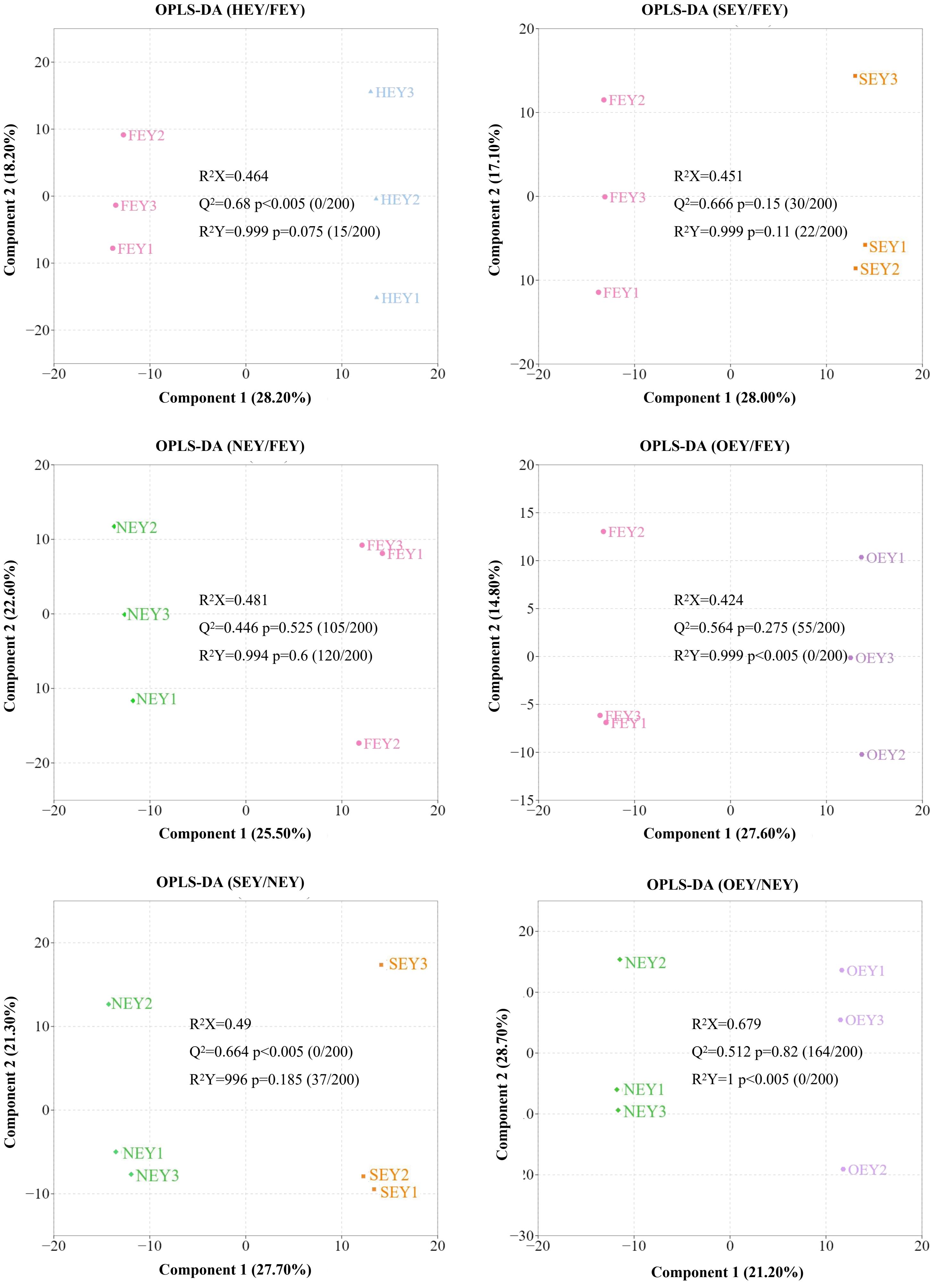

Figure S3: Orthogonal partial least squares discriminant analysis (OPLS-DA) of the lipid profile of egg yolk heat-treated at different intensities.

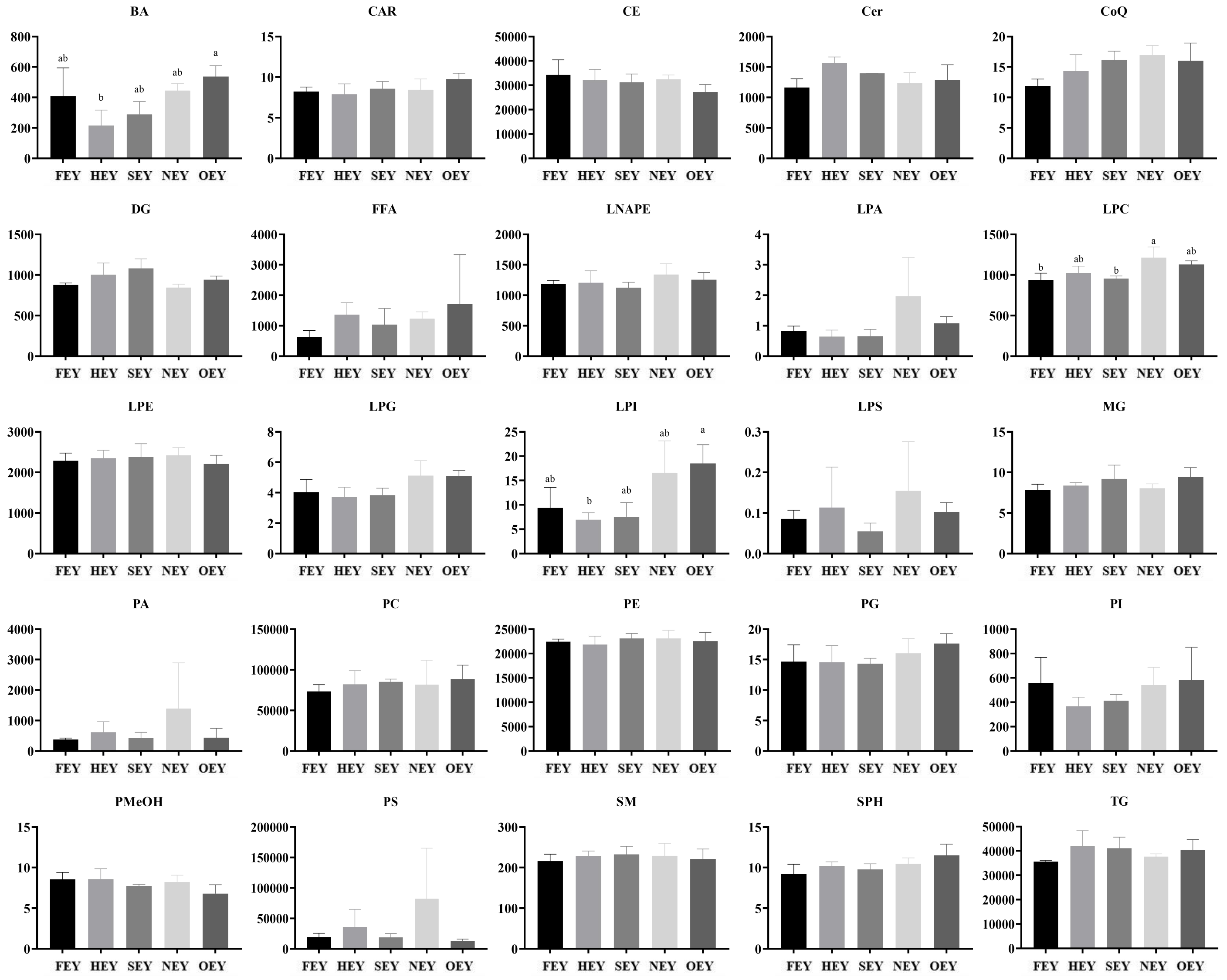

Figure S4: Changes in the abundance of lipid categories in egg yolk with different intensity of heat treatment.

Figure S5: Number of double bonds contained in differentially abundant TG molecules in HEY, NEY, and OEY (A, HEY/FEY; B, NEY/FEY; C, OEY/FEY).

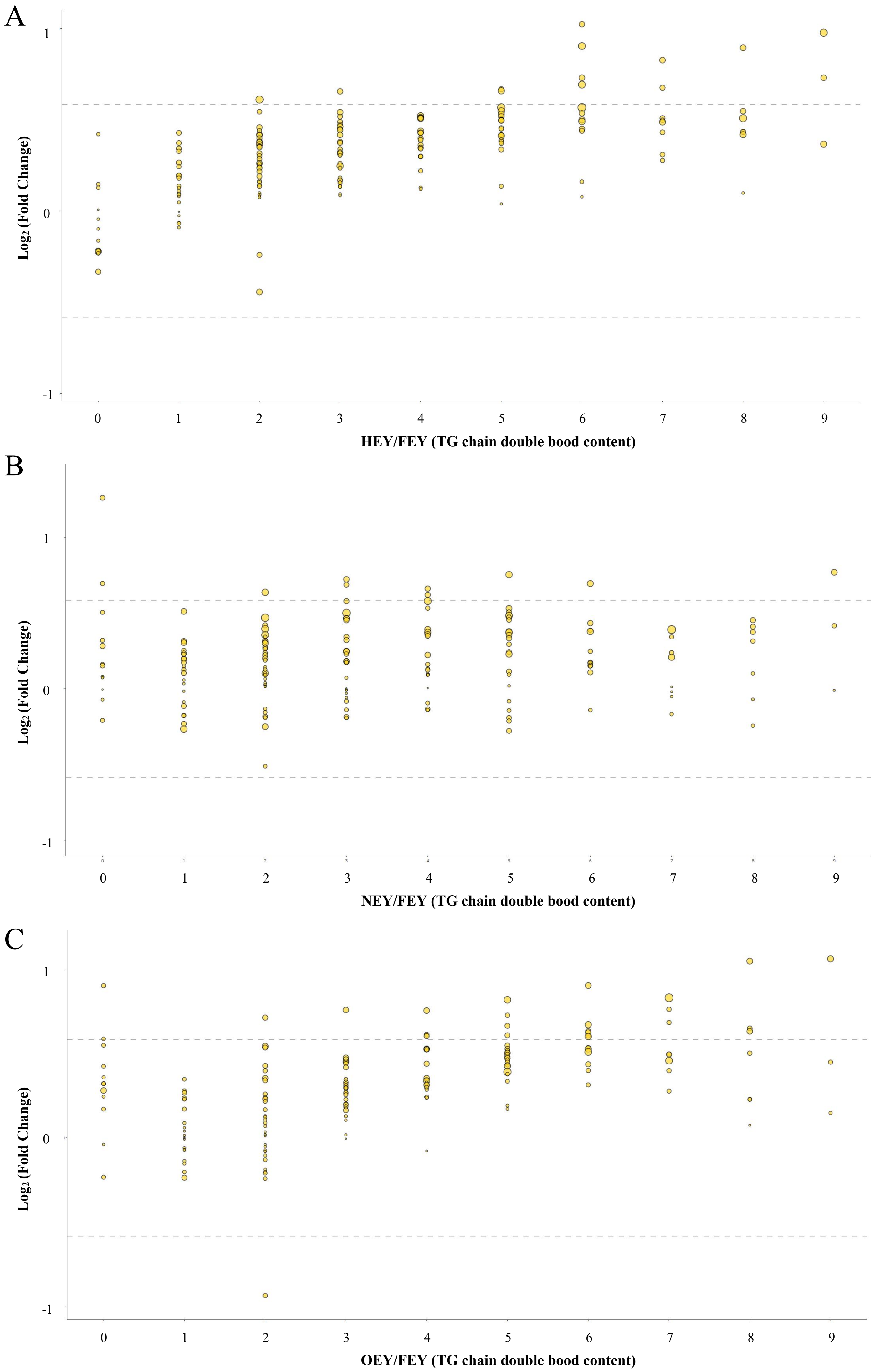
A
Log2 (Fold Change)
1
0
-1
0
1
2
3
4
5
6
7
8
9
HEY/FEY (TG chain double bood content)
B
Log2 (Fold Change)
1
0
-1
0
1
2
3
4
5
6
7
8
9
NEY/FEY (TG chain double bood content)
C
Log2 (Fold Change)
1
0
-1
0
1
2
3
4
5
6
7
8
9
OEY/FEY (TG chain double bood content)
